# Successful Eradication of the Asian Longhorn Beetle, *Anoplophora glabripennis*, from North-Eastern Italy: Protocol, Techniques and Results

**DOI:** 10.3390/insects12100877

**Published:** 2021-09-28

**Authors:** Matteo Marchioro, Massimo Faccoli

**Affiliations:** Department of Agronomy, Food, Natural Resources, Animals and the Environment, University of Padua, 35122 Padova, Italy; massimo.faccoli@unipd.it

**Keywords:** Cerambycidae, invasive pest, eradication program, ALB

## Abstract

**Simple Summary:**

*Anoplophora glabripennis* (Coleoptera: Cerambycidae) is an extremely polyphagus Asian wood-boring beetle accidentally introduced into North America and Europe. In 2009, an infestation was found in the municipality of Cornuda (Veneto Region, Italy). In order to eradicate the pest, several actions were immediately undertaken in the delimitated infested and buffer zones: tree visual inspections twice a year, felling and chipping of infested and suspected trees, trapping protocols, mitigation plans based on substitution of felled trees with new plants, and citizen alerts. The program lasted 11 years, after which the species was declared eradicated from the region. During the eradication program more than 36,000 trees were surveyed and more than 2000 trees were felled. Trees most affected by the pest were birches, elms, maples, and willows. This paper describes all the actions undertaken during the eradication program, providing a protocol that can also be used for future eradications of the species.

**Abstract:**

The Asian Longhorn Beetle (ALB), *Anoplophora glabripennis* (Coleoptera: Cerambycidae), is an important and extremely polyphagous wood-boring beetle native to Asia. In the 1990s, ALB was accidentally introduced into North America and Europe. In 2009, a large ALB infestation was found in the Veneto Region (north-eastern Italy), in the municipality of Cornuda (Treviso province). Eradication actions were immediately undertaken, based on delimitation of infested and buffer zones, tree visual inspections, felling and chipping of infested trees, trapping protocols, and citizen alerts. A total of 36,361 trees, belonging to 16 genera, were surveyed twice a year over an area of 7594 hectares. In 2020, after 11 years of eradication measures, the ALB population of Cornuda was declared eradicated. Overall, 2361 trees belonging to 8 genera were felled and destroyed, of which 1157 were found to be infested by ALB. This paper describes all the actions carried out and the procedures applied in order to eradicate ALB from north-eastern Italy, providing a useful example for current and future ALB eradication programs.

## 1. Introduction

The Asian Longhorn Beetle (ALB), *Anoplophora glabripennis* (Motschulsky) (Coleoptera Cerambycidae), is a wood-boring beetle native to China and the Korean Peninsula [1]. Although in its native range *A. glabripennis* mainly infests *Populus* spp., *Salix* spp., *Ulmus* spp., and *Acer* spp. [2], *A. glabripennis* is an extremely polyphagous pest able to develop on woody broadleaves of 34 tree species belonging to 14 genera in 10 families [1,3]. *A. glabripennis* attacks both young and mature trees growing in urban and peri-urban areas [3,4,5]. Unlike most longhorn beetles, *A. glabripennis* develops in apparently healthy plants, although it can also infest stressed trees and fresh logs [6,7]. For these reasons, the introduction of *A. glabripennis* in new areas represents an enormous threat to urban parks and rural forests [8,9].

Accidentally introduced with infested wood packaging material associated with international trade [3], *A. glabripennis* was first found outside its native range in New York City (NY, USA) in 1996 [10,11]. Following this, the pest was found also in other states of the USA (Illinois, New Jersey, Massachusetts, Ohio, and South Carolina) [4,12,13,14,15], in Canada (Ontario) [16], and in Europe, where the first presence of *A. glabripennis* was recorded in Austria (2001) [17], followed by France (continental) (2003) [18], Germany (2004) [19], Italy (2007) [20], the Netherlands (2010) [21], Switzerland (2011) [22], the UK (2012) [23], Corse (2013) [24], Finland (2015) [25], and Montenegro (2015) [26]. After eradication measures undertaken by different states, to date, infestations of the pest still occur in France (both continental and Corse), Germany, Italy, and the USA [27].

In Italy *A. glabripennis* was found in the northern regions of Lombardy (2007) [20], Veneto (2009) [28], Piedmont (2018) [29], and in the central region of Marche (2013) [30]. At these latitudes the whole *A. glabripennis* life cycle lasts 12–18 months (Faccoli, pers. observ.). Adults emerge in summer, mainly in late June–early July [31]. After maturation feedings and mating, the female lays eggs in oviposition pits chewed out under the bark of the host tree [31]. Young larvae initially feed on phloem and, starting from the third instar, they complete their development in the wood [32]. Pupation lasts a couple of weeks, in a pupal chamber created by mature larvae in the sapwood, and the new adult emerges through a circular hole (about 10 mm diameter, smaller in males) [32]. In Europe, complete development of *A. glabripennis* has been recorded on many genera of woody broadleaves.

The *A. glabripennis* infestation occurring in north-eastern Italy (Veneto Region) was discovered in June 2009 in the municipality of Cornuda [28,33]. A maple was found to be infested in a private garden of the city center following a report of the garden owner to the local phytosanitary office of the Regional Plant Protection Organization (RPPO). The infested tree showed all the typical symptoms of *A. glabripennis* infestation, with large and circular emergence holes, sectorial dieback of the canopy with dead branches, and oviposition pits along the stem. Immediately after the discovery of the pest, a large-scale intensive monitoring and eradication program started. The eradication program was based on the establishment of buffer zones, visual inspections, felling and destruction of infested trees, trapping protocols, and citizen alerts, following the official guidelines issued by EPPO [34]. The *A. glabripennis* population of Cornuda was declared eradicated in 2020 [35], after 11 years of application of specific control measures. The aim of this paper is to present and describe all the actions and procedures successfully carried out in order to eradicate *A. glabripennis* from north-eastern Italy.

## 2. Materials and Methods

### 2.1. Site Description

The municipality of Cornuda (45°830′ N, 12°010′ E; Italy), is located along the southern border of the Italian Alps, approximately 160 m a.s.l., in a transition from continental to Mediterranean climatic conditions, with temperate summers and winters. Annual precipitation ranges from 1100 to 1200 mm, concentrated in the spring and autumn. Cornuda is a small village of about 6200 inhabitants located in a suburban area fallowing within a hilly landscape with patches of agricultural land. The village structure consists mainly of small, isolated houses surrounded by small private gardens often containing ornamental trees represented by woody native or exotic broadleaves. Public tree-lined parks and rows of trees along the streets are also common.

The village is closely surrounded by mixed deciduous forests and riparian habitats which develop along the Piave river. Natural forests are primarily composed of *Acer pseudoplatanus* L., *Carpinus betulus* L., *Fagus sylvatica* L., *Fraxinus excelsior* L. and *Quercus robur* L. on shady damp slopes, as well as *Betula pendula* Roth., *Fraxinus ornus* L., *Ostrya carpinifolia* Scopoli and *Quercus pubescens* Willd. on sunny dry slopes.

### 2.2. Tree Database

Since the first record of the *A. glabripennis* in June 2009, an extensive monitoring program started, visually checking one-by-one all of the possible host trees occurring in Cornuda and in the surrounding municipalities (see next paragraphs), with search directions radially oriented from the point of first discovery of the pest. All host trees were also geo-referenced, creating a specific database reporting information about the plant (genera, age, general physiological conditions), geographic position (coordinates), and ownership data (owner name, address and phone number). Some import–export companies work in in the Cornuda territory, with frequent commercial trade with China. As the infestation may have originated from these companies, particular attention was paid to surveying their surrounding areas.

### 2.3. Visual Tree Survey

**Figure 1:**
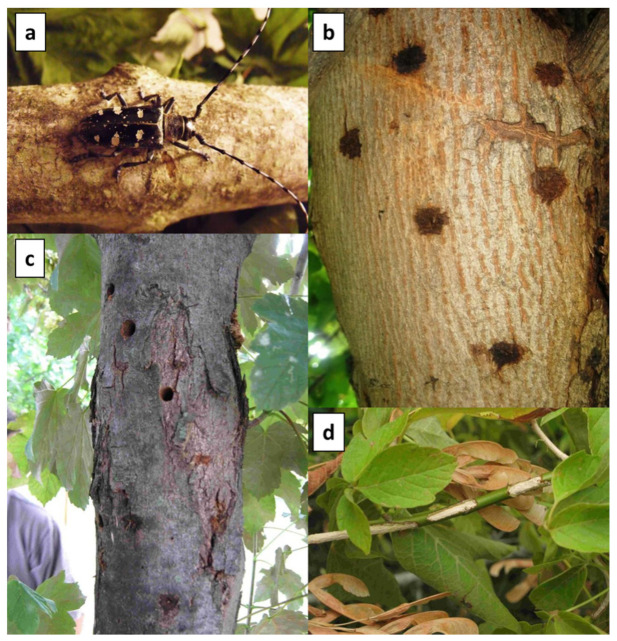
Main symptoms of *A. glabripennis* infestation: presence of adults (**a**), adult exit holes (**b**), oviposition scars (**c**), maturation feedings on twigs (**d**).

After the first survey carried out in summer 2009, with the creation of the tree database, a systemic and continuative survey of the host-plants started, choosing the most susceptible tree genera according to the literature: *Acer*, *Aesculus*, *Alnus*, *Betula*, *Carpinus*, *Cercidiphyllum*, *Corylus*, *Fagus*, *Fraxinus*, *Ostrya*, *Platanus*, *Populus*, *Prunus*, *Salix*, *Tilia* and *Ulmus* [3,4]. The aim of the survey was to discover the presence of *A. glabripennis*-infested plants and update the infested and buffer areas accordingly. The survey was conducted by 3 teams of operators each composed by 2 trained technicians of the Regional Plant Protection Organization that checked all the host-trees one-by-one from the ground, with the use of binoculars looking for *A. glabripennis* infestation symptoms. All host-trees were geo-referenced and checked twice a year, in summer and late-autumn, looking for adult exit holes, larval frass emission, oviposition scars, maturation feedings on twigs, tree decline with branch dieback, and presence of adults on branches and canopies (Figure 1). On one hand, some symptoms are recognizable more easily in summer, such as the presence of recently emerged adults, fresh oviposition pits on the bark, maturation feedings performed by immature adults on twigs, and occurrence of dying branches. On the other hand, the presence of emerging holes is more visible in late autumn after the plants have lost leaves and the upper branches and canopy are easily checkable from the ground. In case of large trees or plants having trunk and branches covered with ivy, difficult to check from ground with accuracy, the inspectors of the Phytosanitary Service were supported both in summer and winter by a team of 4 tree-climbers from the Treviso Forest Service, who also checked for the potential presence of symptoms in the upper part of the canopy. 

Forested areas neighboring the village of Cornuda and falling within the infested or buffer areas were surveyed once per year, along the main forest edges (i.e., external edges, forest tracks, and clear-cut edges). A strip at least 30 m deep inside the forest was monitored. Studies conducted in native regions of South Korea suggest that *A. glabripennis* is not a true forest species but is adapted to riparian habitats characterized by long edges [36]. Similarly, in countries where they are introduced, *A. glabripennis* infestations are usually limited to urban trees that are isolated, growing in small groups or rows, in small rural stands or along forest edges [3,4]. 

### 2.4. Zone Delimitation

**Figure 2:**
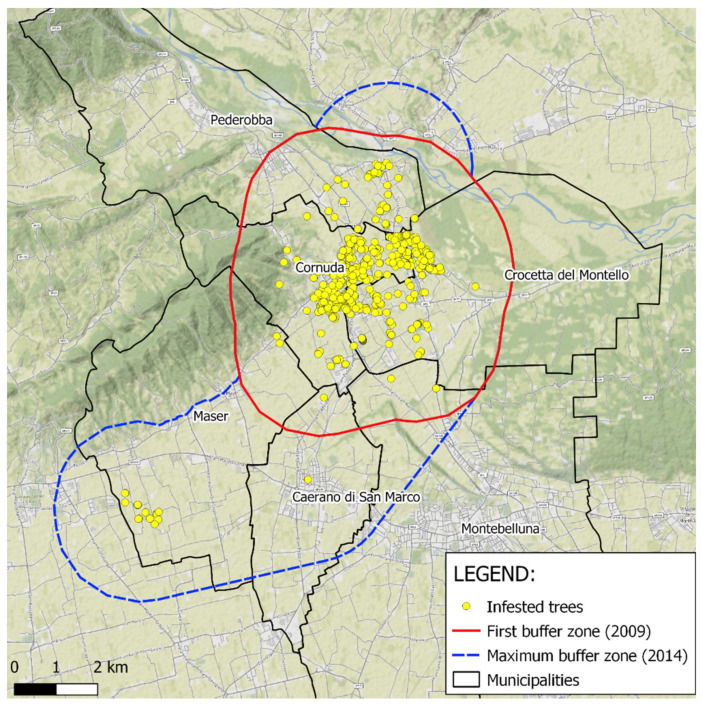
Map of the *A. glabripennis* infestation, with position of infested trees, involved municipalities border and first and maximum buffer zones extension.

After the checking of all host plants growing in the area, the infested and buffer zones were established (Figure 2). The “infested zone” consisted of a polygon including all the infested plants, where the vertices of the polygon were the more external infected plants. The infested zone was included in the territory of six municipalities (Cornuda, Pederobba, Crocetta del Montello, Maser, Montebelluna and Caerano di San Marco). Then, a “buffer zone” was established with a radius of 2 km around the infested zone, i.e., around the most external infested trees, according to European regulations [34]. Year-by-year, when new infested trees were found, the delimited zones (infested and buffer zones) were officially extended (or restricted) several times, coming to also include the bordering municipalities where satellite infestations have been discovered since 2010. 

### 2.5. Pheromone Traps

During the eradication program, trapping protocols were carried out in the buffer zone in order to verify the presence of *A. glabripennis* in the territory and to test the effectiveness of different trapping tools (i.e., trap models and lures). The sites for trap-setting were chosen in relation to the concentration of susceptible hosts and in suitable areas near the edge of the delimited zone. 

In 2011, 14 traps of different type, size, and color were placed for one month (August) and checked weekly (Table 1). Traps were baited with a blend produced by ChemTica International (Heredia, Costa Rica). In 2012, 27 black cross-vane traps were set up and baited with either the ChemTica blend used in 2011, or a Russian experimental blend provided in six different formulations by Dr. Oleg Kulinich of the Department of Forest Quarantine, of the All-Russian Center of Plant Quarantine of Moscow. Traps were checked three times during July, the month with the highest emergence of *A. glabripennis* adults [31]. In 2013, 24 traps were placed: 6 control unbaited traps, and 6 others for each of three different blends, equally divided (ChemTica blend and two new formulations of the Russian blend). Lastly, in 2019, ten black cross-vane traps were set in the delimited zone and checked every 2 weeks from middle June to middle September, in order to support the action of visual inspections and verify if *A. glabripennis* was successfully eradicated. Traps were baited with the ChemTica blend tested in the previous year. The used trap types and lure formulations are summarized in Table 1. 

### 2.6. Sanitation Felling and Tree Destruction

All trees detected during the visual survey as showing *A. glabripennis* infestation symptoms were cut and destroyed (details below). Trees reporting unclear symptoms possibly confused with those potentially caused by others urban pests infesting the same host-trees (e.g., *Cossus cossus* (Lepidoptera: Cossidae), *Zeuzera pyrina* (Lepidoptera: Cossidae), *Saperda carcharias* (Coleoptera: Cerambycidae)) were cut and destroyed as well.

*A. glabripennis* adults leave the tree where they emerged only rarely—when they are strongly disturbed, for example, by tree felling. Moreover, adults were found to be active from the end of May to November. Therefore, infested trees found during the spring–summer survey were marked but not cut immediately, to avoid adult dispersal during tree felling and movement of infested wood through the village. Tree felling, carried out by workers of the Regional Forest Service, was hence postponed every year to winter, between December and April, during insect hibernation as mature larvae [31]. Infested trees, from both public and private properties, were felled at ground level, leaving only stumps, and moved to a fenced and paved storage area falling within the infestation zone, but away from host plants. In winter, cut trees were then chipped in 2-cm long chips, and chips sold to a biomass power station outside the infestation area. Wood chips have been submitted to entomological analysis by the University of Padua in order to exclude the presence of live larvae. However, chips were moved to the biomass power station and burned by the end of winter to reduce the risk of dispersal outside the infestation area of woody material potentially infested with active *A. glabripennis* stages.

The first 4 years of eradication (2009–2012), tree cutting, and chipping only concerned infested plants and a few infested trees growing close to the attacked ones. Then, in order to make the eradication protocol more effective, the Regional Decree no. 33 of 10 September 2012 of the Veneto Region introduced the “Clear-cut” measure, which involved identification and cutting of all susceptible plants, even if apparently uninfested, occurring in the area within a 50 m radius from each infested plant. Since 2015, considering the imminent approval of the Decision UE/2015/893 of the European Commission, the clear-cut radius has been increased to 100 m. 

### 2.7. Mitigation Plan

In accordance with the owner’s will, plants felled in private gardens were replaced for free with new trees belonging to non-host species. Young trees (3–4 years old) were chosen by the citizens among the species available for reforestation and urban design programs at the forest nursery of the Regional Forest Service, including *Cercis siliquastrum*, *Clerodendrom trychotomum*, *Ginko biloba*, *Liquidambar styraciflua*, *Quercus robur*, and *Quercus pubescens*. 

### 2.8. Communication

Municipalities and other territorial authorities, such as schools, park administrators and citizens’ associations, were immediately involved. Public meetings were organized to inform citizens, with the distribution of informative brochures and poster hanging, and releases were sent to local newspapers. Moreover, specific technical meetings were organized to inform and train local nurserymen, pruners, gardeners, and other professional stakeholders. Lastly, brief lessons were organized for students of primary and secondary schools of the territory, with projection of slides and photos concerning pest biology and symptoms. In this way, citizens were informed about the threat and how to recognize and report signs of the pest presence. A toll-free number was also activated to allow citizens to report suspect symptoms or *A. glabripennis* sightings. Finally, an internet site was activated providing information for citizens and a platform to upload reports.

## 3. Results

The eradication plan started in the summer of 2009 and ended in 2020 when, according to Commission regulation EU/2015/893, the species was declared eradicated from Cornuda and its surrounding municipalities [35], following four years of no new records of infested plants. Indeed, neither insects nor plants showing infestation symptoms (oviposition pits, emerging holes, or maturation feedings) have been found since 2016.

### 3.1. Visual Tree Survey 

Since the beginning of monitoring in 2009, more than 36,000 trees were surveyed singly over twelve years (Table 2). Among the 16 surveyed genera, the most abundant were *Acer* (10,277 trees, 28%), *Ulmus* (6227 trees, 17%), *Salix* (5271 trees, 15%), *Carpinus* (4837 trees, 13%), and *Betula* (2067 trees, 6%); other genera occurred in percentages lower than 5% (Table 2). 

During the survey carried out from 2009 to 2020, 1157 trees belonging to 8 genera were found to be infested (3% of the total amount of surveyed trees). The most at-tacked genera were *Acer* (431 trees infested), *Ulmus* (337 trees infested), *Betula* (210 trees infested), and *Salix* (149 trees infested)—even if, looking at the ratio between in-fested and monitored trees, the most susceptible genera were *Cercidiphyllum* (18.2%), *Aesculus* (11.6%) and *Betula* (10.2%), followed by *Ulmus* (5.4%), *Acer* (4.2%), and *Salix* (2.8%) (Table 2). Monitored but never found infested genera were Alnus, Carpinus, Cor-ylus, Fagus, Fraxinus, Ostrya, Platanus and Tilia (Table 2).

### 3.2. Zone Delimitation 

In 2009, the year of the infestation discovery, the delimited zone had an area of 4105 ha which grew in the subsequent years, reaching the maximum size of 7594 ha in 2013 (Figure 2). In 2016, as a result of the application of the eradication protocol, both infestation and buffer zone began to decline, reaching the minimum value of 1843 ha in 2018 (Table 3).

### 3.3. Pheromone Traps 

During the three year monitoring with traps (2011–2013), only two *A. glabripennis* females were caught in 2013. The two individuals were caught by one multi-funnel and one cross-vane trap, both baited with the ChemTica blend. In 2019, after three years without finding any attacked plants, traps were placed to provide further confirmation that eradication had taken place, and no individuals were caught.

### 3.4. Sanitation Felling, Tree Destruction, and Mitigation Plan 

Beside the 1157 infested trees, another 1204 trees were felled because they were inside the “clear-cut area” (Table 3). Overall, a total of 2361 trees were cut in 12 years during the eradication plan applied in Cornuda, of which only 220 plants were from private gardens. The highest peak of felling occurred during the first year (2009), with 630 cut trees. Subsequently, the number was steadily decreasing with time, until no new plants were felled since 2016.

Two samples of wood chips, taken in 2010 and in 2011, have been submitted to an entomological analysis by the University of Padua. The analysis showed that the size of the wood pieces from the chipping operations is incompatible with the development and survival of *A. glabripennis* larvae in the wood; almost all of the material analysed was less than 2 cm in length, compared to 4–5 cm in length for mature larvae. In fact, several remains of crushed larvae were found during the analysis. Moreover, the few larger elements are subject to rapid deterioration due to tissue dehydration or fermentation, depending on the humidity conditions. In conclusion, the product tested was found to be biologically safe and free from risk of spreading *A. glabripennis* infestation. 

Main costs incurred in carrying out eradication program were:

- €380,000 used by the Regional Plant Protection Organization for the annual tree survey (twice per year).
- €520,000 used by Regional Forest Services for felling and chipping trees.
- €20,000 used by the University of Padua for scientific support and research activities.

The program was initially financed by funds from the Veneto Region, which then accessed European funds for the management and eradication of invasive species.

As compensation for the felling of infested trees occurring in private properties, owners could choose a new tree to plant as a replacement. A total of 217 new trees (over 220 cut) were planted, including *Cercis siliquastrum* (65), *Liquidambar styraciflua* (41), *Ginko biloba* (35), *Clerodendrom trychotomum* (29), *Quercus robur* (27), and *Quercus pubescens* (20). There was no financial compensation.

## 4. Discussion

Eradication is the numerical reduction of a population in a specific geographic area to prevent its reproduction and, therefore, bring it to local extinction [37,38]. Conditions that support a higher probability of successful eradication include early detection of the pest (i.e., limited spatial distribution), ability to detect and identify the invader or the infested plants, availability of effective tools for pest monitoring and control, and public support [39,40]. Moreover, the target species should have all or most of the following characteristics: low rate of reproduction and dispersal, ease of detection at low population density, and limited host range [39]. 

Early detection of the pest plays a key role in a successful eradication program. The earlier the parasite is discovered from the time of actual arrival, the higher the chances of success, as the parasite will have less time to reproduce and spread. In fact, the probability of successful eradication declines with increases in the infested area [41,42]. In particular, Rejmánek and Pitcairn [42], analyzing data from 53 infestations of 18 pest species, showed that eradication success probability is about 50% between 0.1 and 1.0 hectares and about 25% between 100 and 1000 hectares of infested area. Moreover, as the area of eradication increases, the required efforts (i.e., costs) also increase and the operation may no longer be economically viable [40]. The Cornuda infestation initially measured about 4000 ha (infested and buffer zones) and expanded to a maximum extension of about 7600 ha. Although the infestation was discovered in 2009, it was verified, by dating the exit holes from the host trees, that the infestation started at least five years previously, in 2005 [31]. This delay in starting the eradication program caused an effort of eight years of active eradication (2009–2016) and another four years (2017–2020) of surveying in order to eradicate *A. glabripennis* from the territory. The monitoring protocol involved more than 36,000 trees checked one-by-one twice a year for 11 years; 2361 of these trees were felled because they were found to be infested or simply because they were within the clear-cut radius. Another example of successful *A. glabripennis* eradication is at Paddock Wood (Kent, UK), although in this case the infestation was much smaller (with an infested zone of only 11.4 ha). After just one year (and another seven years of surveying) the pest was eradicated and about 2200 trees were felled, of which 66 were infested [43,44]. In contrast, a large infestation was detected in Worcester (MA, USA) in 2008 [5], and is still active [45]. Until 2015, the extension of the infestation was larger than 20,000 ha with more than 5 million monitored trees, of which approximately 34,000 were removed (both infested, and those deemed to be high-risk) [46]. Such a wide spread makes a successful eradication challenging [5].

Besides early detection, a successful eradication is based on the possibility of easily identifying the pest or its infestation symptoms and the disposal of effective tools for its detection. Visual inspections have proved to be effective against *A. glabripennis*, but they lose effectiveness for recently infested trees [43], or in the case of large trees or trunks covered by ivy (Faccoli, pers. observ.). Pheromone traps are often used alongside the work of phytosanitary inspectors, both to find pests and for their eradication by mass-trapping and lure-and-kill techniques [47,48,49,50]. Unfortunately, no long-range pheromone has been reported for *A. glabripennis*, although both male-produced short-range and female-produced contact recognition pheromones have been identified [3,4,51,52]. Several studies have tested the effectiveness of these pheromones combined with some host-volatiles (e.g., Z-3-hexen-1-ol and Linalool) in trapping protocols, showing some positive outcomes, but with few catches despite the dozens of traps used [53,54,55]. During the eradication program carried out in Cornuda, only two *A. glabripennis* females were caught by traps in 2013 and no *A. glabripennis* individuals were caught by traps used at Paddock Wood [43]. Despite the use of *A. glabripennis* pheromones remaining indicated for pest interception in areas where it is not yet been detected [56], our results corroborate the hypothesis that the attraction of pheromones is not strong enough to be used for active eradication actions by mass-trapping, and probably not even for reliable monitoring. The low effectiveness of pheromone-based trapping techniques is probably due to the fact that they mainly attract virgin females and, at close range, females also used other visual and chemical stimuli which require further study [57]. 

Despite *A. glabripennis* having many of ecological and biological characteristics indicated by Brockerhoff et al. [39] as facilitating their eradication, an effective *A. glabripennis* eradication is never easy because of its extreme polyphagy and the generic symptoms it causes to host plants. First of all, *A. glabripennis* has a low fecundity rate [3]. In China, under natural conditions, 25–40 viable eggs were estimated per female [4], whereas in the USA that fecundity was estimated to vary between 30–178 eggs per female [58,59]. Additionally, their limited active dispersal capacity is an important factor. The potential dispersal of *A. glabripennis* adults was estimated at about 2000 m, with a realistic annual spread of about 300 m from the closest infested tree [56,60,61]. Moreover, the tendency to reinfest the same tree for several years was usually observed [3,4]. Lastly, according to climatic conditions *A. glabripennis* takes 1–3 years or even more to fully complete its life cycle [3,4]. All these characteristics (i.e., fecundity, active fly, and life cycle duration) strictly depend on temperature [62]. In Cornuda, annual temperatures range between −2 and 29 °C, with a mean temperature of 23 °C during warmer months [63]. In Paddock Wood annual temperatures vary between 2 and 23 °C, with a mean temperature of 17 °C during warmer months [64]. The first effects of the different climatic conditions at the two sites can be observed on the life cycle. In northern Italy *A. glabripennis* was considered univoltine [61], whereas for the UK a 2–3 year life cycle was estimated [64]. Moreover, research carried out on the effects of temperature on *A. glabripennis* fecundity estimated that the optimum temperature for maximum fecundity is about 25 °C [59]. Another study showed that the adult’s flight capacity increases with temperatures from 15 to 30 °C, and that no flight occurs under 15 °C [65]. In conclusion, lower temperatures of Paddock Wood caused a lower *A. glabripennis* adult fecundity, lower flight propensity (i.e., lower dispersion of the infestation), and lengthening of development time—doubling or even tripling it compared to the Cornuda infestation. All these factors probably contributed to keeping the pest infestation low in the UK, despite the eradication beginning about ten years after the estimated arrival of the pest [66], whereas in Italy only five years had elapsed.

One of the major problems in dealing with *A. glabripennis* eradication concerns its extreme polyphagy on woody broadleaves. An extensive investigation conducted in the Yinchuan region (China) found damage on trees belonging to 14 genera of broadleaves, although complete development has not been confirmed on all species listed as hosts [4]. However, host suitability differs in different continents: *Populus* and *Salix* are more suitable than *Ulmus* in China [4]; *Acer* and *Ulmus* are generally more suitable than *Fraxinus* in the USA [15,67]; in Europe the most suitable genera are, in decreasing order, *Acer*, *Betula*, *Salix*, *Aesculus* and *Populus* [18]. In the Cornuda infestation the most infested genera (by percentage) were *Betula*, *Ulmus*, *Acer*, and *Salix* (excluding *Cercidiphyllum* and *Aesculus* because of the small number of trees present)—similar to infestations in other Italian regions (Lombardy, Marche, and Piedmont) [35] and to the infestation at Paddock Wood, where the most-attacked genera were *Acer*, *Salix*, and *Betula* [43,44]. Interestingly, in both the Cornuda and Paddock Wood infestations, the number of infested poplars was very low; this is particularly evident in Italy, were only 2 out of 1709 poplars (0.1%) were found to be infested. In contrast, poplars are among the most suitable hosts for *A. glabripennis* in China, even if not all Populus species are equally susceptible to *A. glabripennis* [68]. *Acer*, instead, is confirmed as one of the main hosts for *A. glabripennis*. Such a large polyphagy has important consequences in the management of *A. glabripennis* infestations. A higher number of potential hosts means, on one hand, higher chances for the pest to reproduce and proliferate and, on the other hand, a higher number of trees to be surveyed and, if infested, to be felled and replaced. Moreover, *A. glabripennis* infests healthy and vigorous trees [3,4], which makes prevention more difficult, as keeping plants healthy and in good physiological condition does not prevent infestations.

The eradication of *A. glabripennis* from the municipality of Cornuda shows the importance of taking prompt, coordinated, and effective actions to contain the spread of the pest and to proceed with its systematic elimination from the infested area. Despite the considerably large size of the Italian infestation, the benefits obtained from the eradication of the pest have far exceeded the costs incurred for its implementation [69]. Of course, different scenarios may occur. For example, the *A. glabripennis* infestation occurring in Worcester (Massachusetts) seems to be too widespread now and the eradication, although it remains the goal, is of uncertain outcome and is taking enormous effort [46]. 

Of all the actions undertaken, those that proved the most effective were visual survey of susceptible trees (in order to find signs of infestation) and the felling and destruction of all the infested trees and nearby ones (in order to prevent the diffusion of the pest). Additionally, the destruction of felled trees by chipping is a very useful practice in order to kill all the larvae present inside the wood, as demonstrated by the wood chip analyses conducted by the University of Padua and already reported in other works [57,70]. On the other hand, the use of pheromone traps proved useless, as was also the case during the eradication in Paddock Wood [43]. Finally, the involvement and active participation of citizens and stakeholders is also of paramount importance [39,71]. If not properly motivated, ‘unpopular’ actions such as felling private trees and killing insects may lead to protests and non-cooperation by the population, jeopardizing the outcome of the whole operation. In this respect, the first report of the presence of *A. glabripennis* in Cornuda was made by a private citizen, demonstrating the importance of citizens’ cooperation in the interception of alien species.

## Figures and Tables

**Table 1:** Information about detection tools used during the eradication program. For each year information was reported about the trap model (type and color), the trap number, and the type of lure.

Year	Trap Type	Trap Color	Trap Number	Lure
2011	Cross-vane, long	Black	2	CHEMTICA
	Cross-vane, long	Transparent	1	
	Cross-vane, short	Black	1	
	Cross-vane, short	Transparent	3	
	Multi-funnel, long	Black	3	
	Multi-funnel, short	White	4	
2012	Cross-vane, long	Black	3	CHEMTICA
			4	RUSSIAN (I)
			4	RUSSIAN (II)
			4	RUSSIAN (III)
			4	RUSSIAN (IV)
			4	RUSSIAN (V)
			4	RUSSIAN (VI)
2013	Cross-vane, long	Black	3	CONTROL
			3	CHEMTICA
			3	RUSSIAN (VII)
			3	RUSSIAN (VIII)
	Multi-funnel, long	Black	3	CONTROL
			3	CHEMTICA
			3	RUSSIAN (VII)
			3	RUSSIAN (VIII)
2019	Cross-vane, long	Black	10	CHEMTICA
Blends legend:				
CHEMTICA = 1:1 ratio of 4-(n-heptyloxy)butanal (0.3 mg) and 4-(n-heptyloxy)butan-1-ol (0.3 mg) + (−)-linalool (3 mg) + trans-caryophyllene (3 mg) + (Z)-3-hexen-1-ol (3 mg)				
RUSSIAN (I) = 1:1 ratio of 4-(n-heptyloxy)butanal (5 μL) and 4-(n-heptyloxy)butan-1-ol (5 μL)				
RUSSIAN (II) = 1:1 ratio of 4-(n-heptyloxy)butanal (50 μL) and 4-(n-heptyloxy)butan-1-ol (50 μL)				
RUSSIAN (III) = 1:1 ratio of 4-(n-heptyloxy)butanal (500 μL) and 4-(n-heptyloxy)butan-1-ol (500 μL)				
RUSSIAN (IV) = 1:1 ratio of 4-(n-heptyloxy)butanal (5 μL) and 4-(n-heptyloxy)butan-1-ol (5 μL) + (Z)-3-hexen-1-ol (100 μL)				
RUSSIAN (V) = 1:1 ratio of 4-(n-heptyloxy)butanal (50 μL) and 4-(n-heptyloxy)butan-1-ol (50 μL) + (Z)-3-hexen-1-ol (1000 μL)				
RUSSIAN (VI) = 1:1 ratio of 4-(n-heptyloxy)butanal (500 μL) and 4-(n-heptyloxy)butan-1-ol (500 μL) + (Z)-3-hexen-1-ol (3000 μL)				
RUSSIAN (VII) = 1:1 ratio of 4-(n-heptyloxy)butanal (0.3 mg) and 4-(n-heptyloxy)butan-1-ol (0.3 mg) + (−)-linalool (3 mg)				
RUSSIAN (VIII) = 1:1 ratio of 4-(n-heptyloxy)butanal (0.6 mg) and 4-(n-heptyloxy)butan-1-ol (0.6 mg) + (−)-linalool (6 mg)				

**Table 2:** Number of monitored and infested trees, with relative percentages of infestation, divided by genera.

Tree Genus	Monitored Plants (n)	Infested Plants (n)	Ratio (%)
*Cercidiphyllum*	11	2	18.18
*Aesculus*	147	17	11.56
*Betula*	2067	210	10.16
*Ulmus*	6227	337	5.41
*Acer*	10,277	431	4.19
*Salix*	5271	149	2.83
*Prunus*	1361	9	0.66
*Populus*	1709	2	0.12
*Alnus*	59	0	-
*Carpinus*	4837	0	-
*Corylus*	1238	0	-
*Fagus*	486	0	-
*Fraxinus*	680	0	-
*Ostrya*	43	0	-
*Platanus*	908	0	-
*Tilia*	1040	0	-
Total	36,361	1157	3.18

**Table 3:** Extension of buffer zones, number of monitored and felled trees (divided in “infested” and susceptible trees), and clear-cut radius adopted for each year.

Year	Buffer Zone (ha)	Monitored Plants (n)	Infested Plants (n)	Susceptible Plants (Clear-Cut) (n)	Total Felled Trees (n)	Clear-Cut Radius (m)
2009	4105	12,816	576	54	630	-
2010	5625	20,366	327	198	525	-
2011	7214	24,292	163	82	245	-
2012	7214	24,292	67	679	746	50
2013	7594	25,223	15	83	98	50
2014	7594	30,990	5	52	57	100
2015	7594	36,361	4	56	60	100
2016	4555	24,511	0	0	0	100
2017	4555	24,511	0	0	0	100
2018	1843	13,041	0	0	0	100
2019	1843	13,041	0	0	0	100
Total (n)		36,361 *	1157	1204	2361	

* Healthy trees were monitored every year, so the total is not the sum of monitored trees for each year, but the total amount of monitored trees during the eradication period. Susceptible trees: healthy host-trees growing close to an infested tree. During the first three years, no clear-cuts were applied systematically; however, in the case of polychromic trees, or trees very close to infested ones, or trees damaged by felling-infested trees, felling was also carried out on plants that were not directly infested.

## Data Availability

Not applicable.
